# Treatment of Rheumatoid Arthritis with Traditional Chinese Medicine

**DOI:** 10.1155/2014/528018

**Published:** 2014-06-04

**Authors:** Wen-Yuan Lee, Hsin-Yi Chen, Kuan-Chung Chen, Calvin Yu-Chian Chen

**Affiliations:** ^1^School of Medicine, College of Medicine, China Medical University, Taichung 40402, Taiwan; ^2^Department of Biomedical Informatics, Asia University, Taichung 41354, Taiwan; ^3^Department of Neurosurgery, China Medical University Hospital, Taichung 40447, Taiwan; ^4^School of Pharmacy, China Medical University, Taichung 40402, Taiwan

## Abstract

Rheumatoid arthritis (RA) is a chronic inflammatory disease that will affect quality of life and, working efficiency, and produce negative thoughts for patients. Current therapy of RA is treated with disease-modifying antirheumatic drugs (DMARDs). Although most of these treatment methods are effective, most patients still have a pleasant experience either due to poor efficacy or side effects or both. Interleukin-6 receptor (IL6R) is important in the pathogenesis of RA. In this study, we would like to detect the potential candidates which inhibit IL6R against RA from traditional Chinese medicine (TCM). We use TCM compounds from the TCM Database@Taiwan for virtually screening the potential IL6R inhibitors. The TCM candidate compound, calycosin, has potent binding affinity with IL6R protein. The molecular dynamics simulation was employed to validate the stability of interaction in the protein complex with calycosin. The analysis indicates that protein complex with calycosin is more stable. In addition, calycosin is known to be one of the components of *Angelica sinensis*, which has been indicated to have an important role in the treatment of rheumatoid arthritis. Therefore, calycosin is a potential candidate as lead compounds for further study in drug development process with IL6R protein against rheumatoid arthritis.

## 1. Introduction

According to WHO statistics, 1-2 per two hundred people suffer from rheumatoid arthritis in 2010 [1]. Half of the adults who have been diagnosed with an autoimmune disease in the past ten years are not in full-time work. Autoimmune disease is a form of connective tissue disease that mainly encroaches upon the epitenon synovium and joint. This inflammation can cause joint deformation leading to disability, and the patient will lose some of the joint activity due of joint pain and wear. This inflammation will systematically affect other extra articular tissues, including vascular, skin, muscles, lungs, and heart. People with rheumatoid arthritis may suffer an increase in myocardial infarction (heart attack), the risk of atherosclerosis, and stroke [2, 3]. Other complications could include left heart failure, pericarditis, endocarditis, cardiac valve inflammation, and fibrosis [4].

Nowadays, as increasing number of mechanisms of diseases have been identified [5–10], the researchers detect more and more potential target proteins against each disease, which are useful for drug design [11–15]. Interleukin-6 receptor (IL6R) is important in the pathogenesis of rheumatoid arthritis (RA) [16, 17]. It is an autoimmune disease which principally attacks synovial joints and causes long-term chronic inflammation. Many research results indicate that RA may be an inherent immune response [18]. Half of the risk of RA is thought to be genetic [1] and it has been found to be strongly associated with the major histocompatibility complex (MHC) antigen HLA-DR4 (specifically 0404 and DR0401) and the expression of genes* PTPN22* and* PADI4*. Family history is therefore thought to be an important risk factor [19, 20] as inheritance of the* PTPN22* gene has been shown to double the vulnerability to RA. It is notable that* PADI4* has been identified as the main risk factor in people of Asian descent [12]. First-degree relative prevalence rate is 2-3%, and the concordance of the disease in monozygotic twins is in the region of 15–20% [21, 22]. Smoking is the most significant nongenetic risk factor in the development of the disease [1], and statistical data indicate that smokers are up to three times more likely to develop RA than nonsmokers, especially in men [23]. There is some statistical evidence that moderate alcohol consumption may have a protective value. [24]. Vitamin D deficiency is common in rheumatoid arthritis cases and may have a causal association [25]. Some trials have found that a vitamin D supplement can reduce the risk of RA, while others have not [25].

A study by Mayo Clinic in 2005 indicated that rheumatoid arthritis patients suffered from more than double the risk of heart disease than the general population [26], independent of other risk factors, such as alcoholism, diabetes, high cholesterol, body mass index, and elevated blood pressure. RA mechanisms leading to increased risk are unclear, but the presence of chronic inflammation has been proposed as a contributing factor [27]. More and more effective treatments of protein diseases are being discovered [6, 8, 28–32], and treatments involving traditional Chinese medicine (TCM) methods are also attracting much attention; therefore, potential lead compounds are expected from investigations [28, 33–40].

We used computer-aided virtual drug screening [41] with data from the traditional Chinese medicine Database@Taiwan (http://tcm.cmu.edu.tw/) [42] for the investigation of docking simulation and employed molecular dynamics for the investigation of changes under the static and dynamic conditions to determine natural, effective lead compounds with fewer putative side effects.

## 2. Materials and Methods

### 2.1. Docking and Candidate Screening

The structure of interleukin-6 receptor (IL6R) was derived from human IL6R kinase from the Protein Data Bank (PDB ID: 1N26) [43]. According to UniProt (P08887), the crystal structure of the binding site is located in residues 94–194. We used the Database of Protein Disorder to verify the stability of the structure with the sequence of crystal structure [44].

The investigation is based on Discovery Studio 2.5.5 LigandFit molecular docking method. The small molecules from TCM database could be used to find suitable candidates for the IL6R receptor. All the traditional Chinese medicine small molecules used for screening had been filtered by Lipinski's rule of five [45, 46] and the properties of absorption, distribution, metabolism, excretion, and toxicity (ADMET) [47] in DS 2.5 to rule out potentially toxic derivatives. The binding site was defined by the cocrystallized ligand location in the crystalline structure. All the small molecules for molecular docking were minimized with the smart minimizer setting under the force field of CHARMM [48]. The results of molecular docking are sorted by Dock score, -PLP1, -PLP2, H-bond forming residues, and H-bond quantity. Pi forming residues were also selected from the top twenty.

### 2.2. Molecular Dynamics (MD) Simulation

The stability of protein-ligand complex with candidate compounds was validated using molecular dynamics simulation by GROMACS 4.5.5 [49]. The production of MD simulation time was 5 ns. The GROMACS tool provides an analysis of the MD trajectories. The g_rms program was used to compare structures by calculating the root mean square deviation (RMSD) [50] to observe the changes of the overall structure in the dynamic simulation process compared to a reference structure. The g_gyrate program was used for calculation of the radius of gyration of atomic groups about the *x*-axis, *y*-axis, and *z*-axis, as a function of time. The g_msd program was used to analyze the mean square displacement of proteins, and the g_energy program was used to analyze the potential energy, total energy, kinetic energy, temperature, volume, density, pressure change of pV, and enthalpy. The g_rmsf program was used to determine the flexibility level of a region of a protein by analyzing the root mean square fluctuation (RMSF) of each amino acid. In this study, we also analyze the vector distribution diagrams of eigenvector, distance analysis of hydrogen bond, structure clustering, variation of secondary structure, and Mdmat analysis. In addition, the program, CAVER 3.0 [51], was also used to calculate the import and export pathways for the compound. The CAVER program is based on the Dynamic Map Ensemble (DyME) application program. Dynamic proteins in DyME can be constructed from many different configurations of the polymer. This method calculates the free space of protein using a Voronoi diagram, which is presumably the pathway of a small molecule.

## 3. Results and Discussion

### 3.1. Docking and Candidate Screening

**Figure 1:**
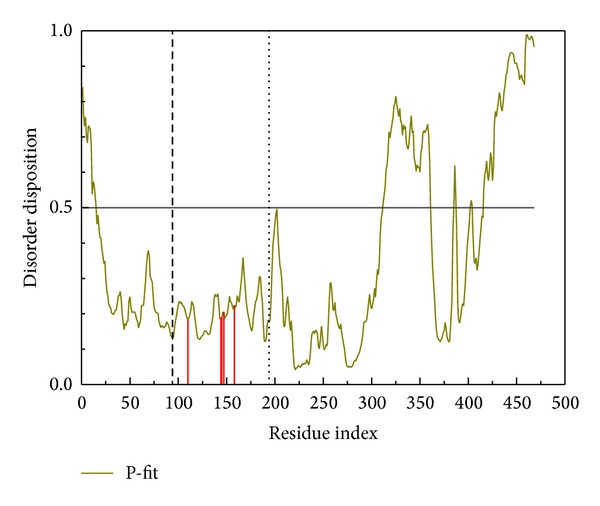
Disordered disposition predicted by PONDR-Fit with the key residues (red line).

Figure 1 shows the results of verification from PONDR-Fit software and the position of important amino acids. As all the important residues in the binding domain are located below the standard line at 0.5, thus the crystalline structure is stable for docking simulation. According to the experimental results (Table 1), the Dock score, -PLP1, -PLP2, H-bond forming residues, H-bond quantity, and Pi forming residues are used to rank the top twenty candidates. Calycosin, the top candidate, is used for further investigation in this paper. In addition, the apoprotein is used as a control.

**Figure 2:**
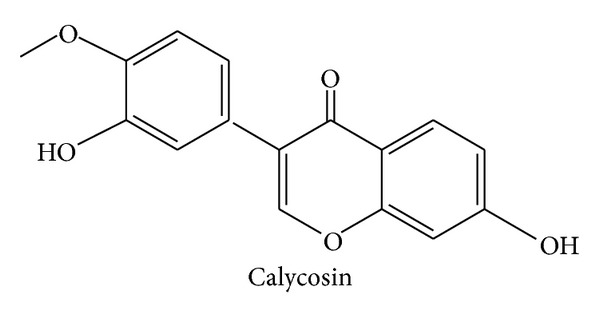
The chemical structure of calycosin.

Recently, plant based drugs have become popular therapies. Since the treatment with plant based drugs had been used thousand years ago, they are thought to be relatively safe and effective drugs [37]. The literature notes [36] that calycosin from Chinese Angelica (*Angelica sinensis*) is a form of complement hematinic false drug. Therefore, we estimated the calycosin content of potential compounds. The structure of calycosin is shown in Figure 2.

**Figure 3:**
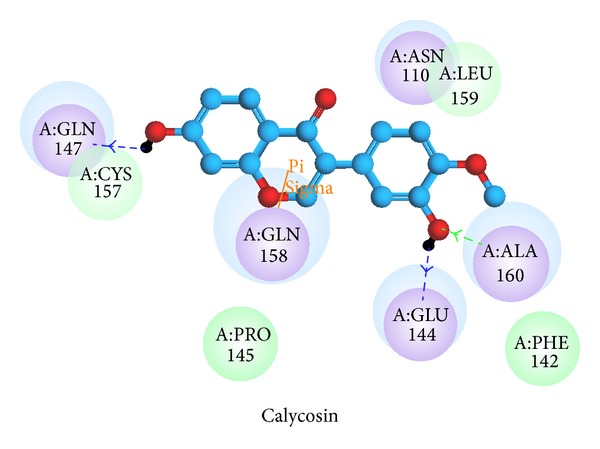
Docking pose of candidate compound in IL6R binding site. Hydrogen bonds are expressed in green and blue dotted lines. *π* bond is shown with an orange line.

**Figure 4:**
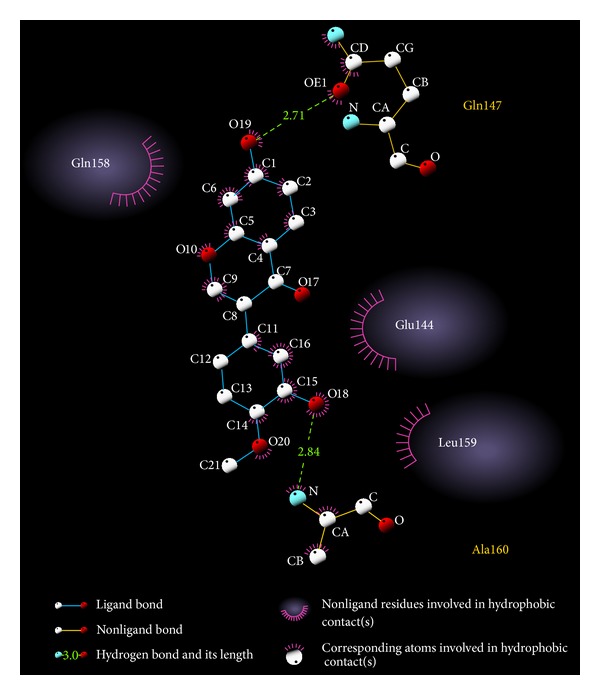
Docking pose of candidate compound in IL6R binding site with hydrophobic contacts.

Figure 3 shows the interactions of the top compound between ligand and residues in binding site. Calycosin has *π* interaction with Gln158, hydrogen bonds with Glu144, Gln147, and Ala160, polarity force with Asn110, Glu144, Gln147, Gln158, and Ala160, and van der Waals force with Phe142, Pro145, Cys157, and Leu159. The stability of calycosin is maintained by the pi interaction, hydrogen bond, polarity, and van der Waals force (Figure 3).  Figure 4 shows the hydrophobic contacts between candidate compound and amino acids in the binding site. Calycosin has hydrophobic contacts with three amino acids, Glu144, Gln158, and Leu159.

According to the docking results in Table 1, calycosin has potent binding affinity with target protein. Due to the results in Table 1, Glu144, Gln147, and Ala160 are important amino acids for binding.

### 3.2. Molecular Dynamics (MD) Simulation

**Figure 5:**
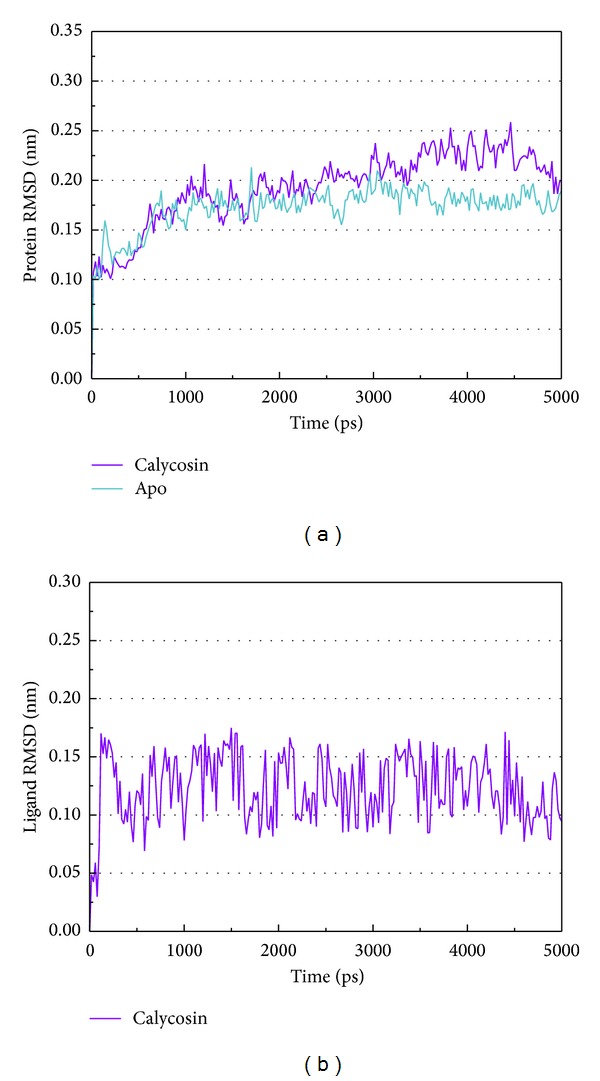
RMSD of protein and calycosin.

**Figure 6:**
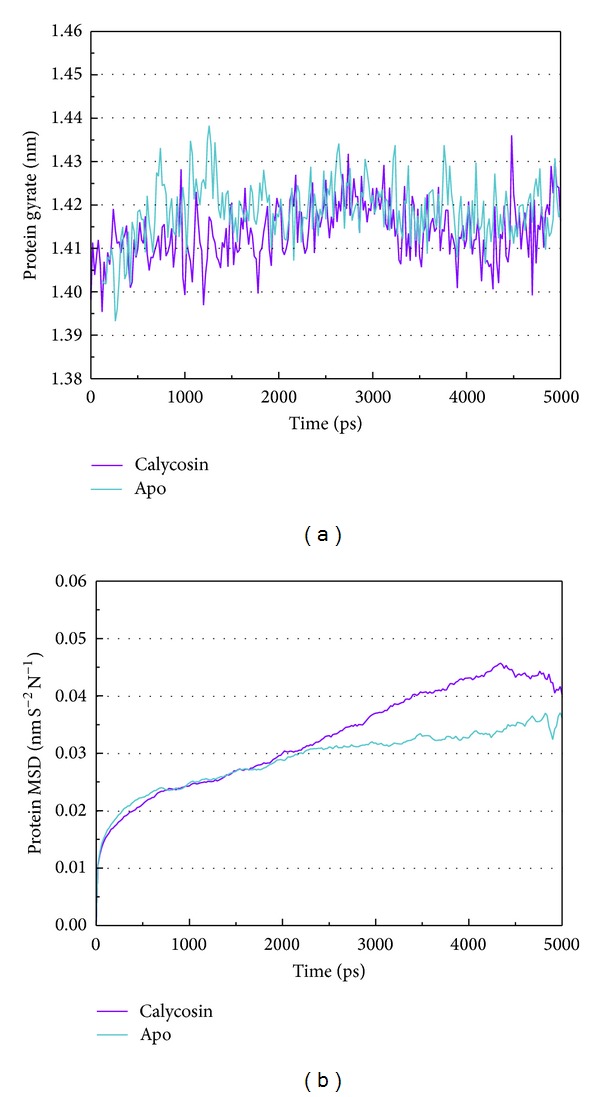
Analysis of MD trajectories generated by Gromacs. (a) Gyrate and (b) mean square deviation (MSD).

**Figure 7:**
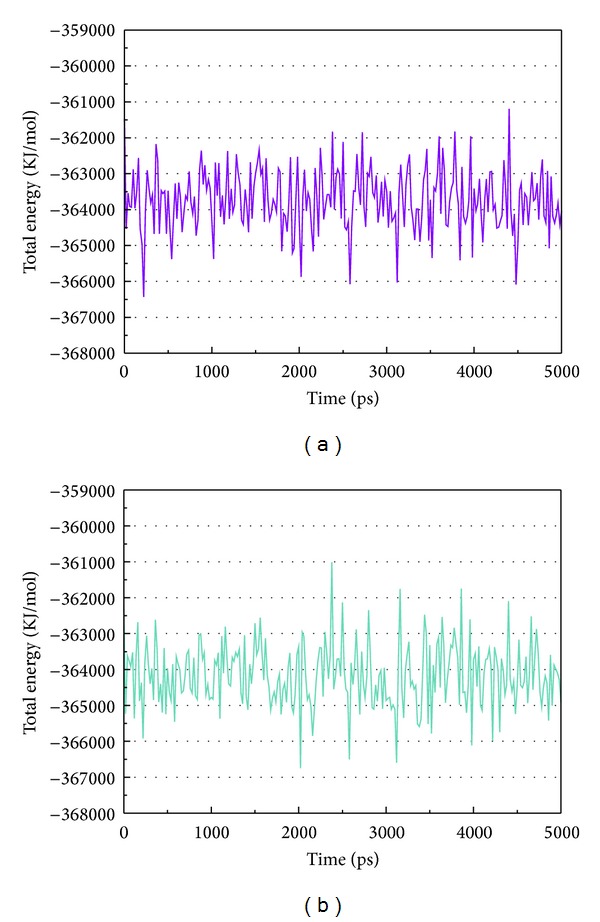
Analysis of transport pathways for (a) protein complex with calycosin and (b) apoprotein.

**Figure 8:**
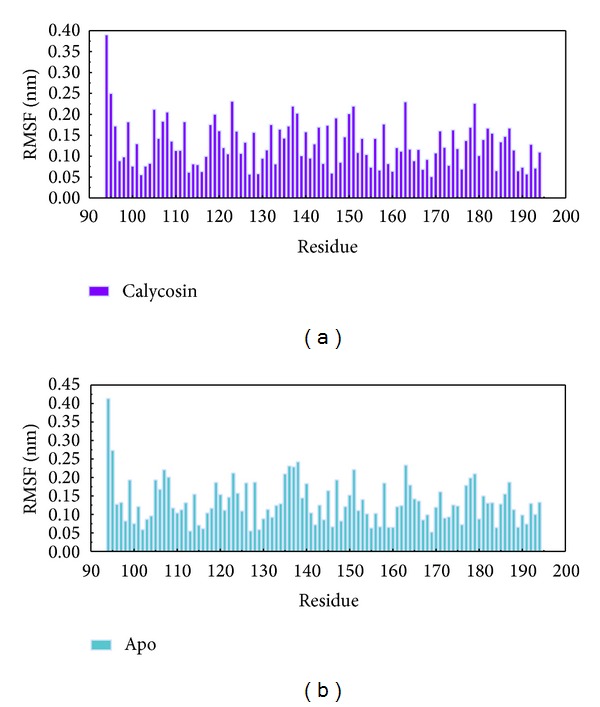
RMSF for residues in (a) protein complexes with calycosin and (b) apoprotein.

**Figure 9:**
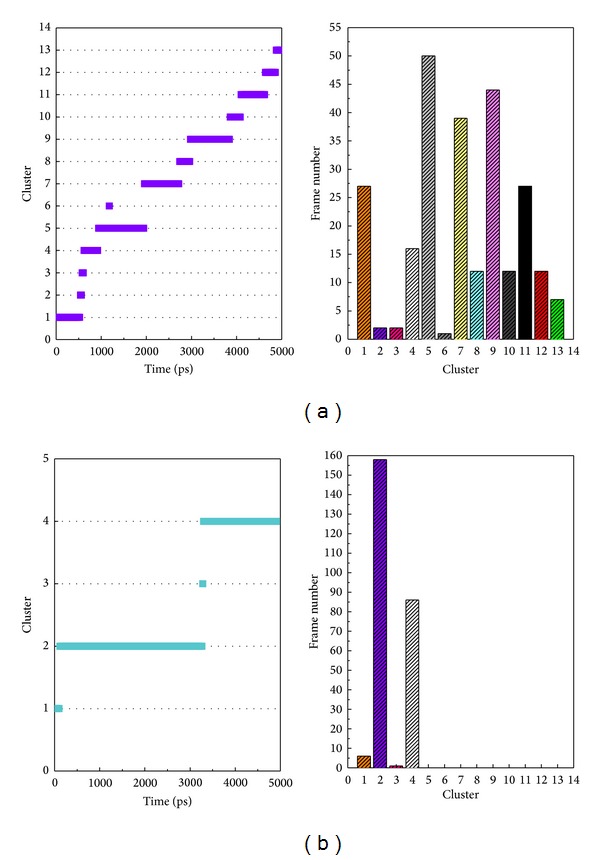
Cluster trend of (a) protein complexes with calycosin (cutoff: 0.142 nm) and (b) apoprotein (cutoff: 0.145 nm).

**Figure 10:**
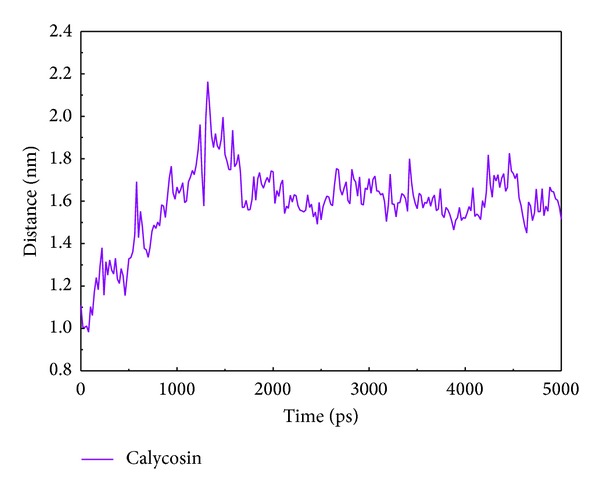
Distance trend between mass centers of protein and calycosin.

**Figure 11:**
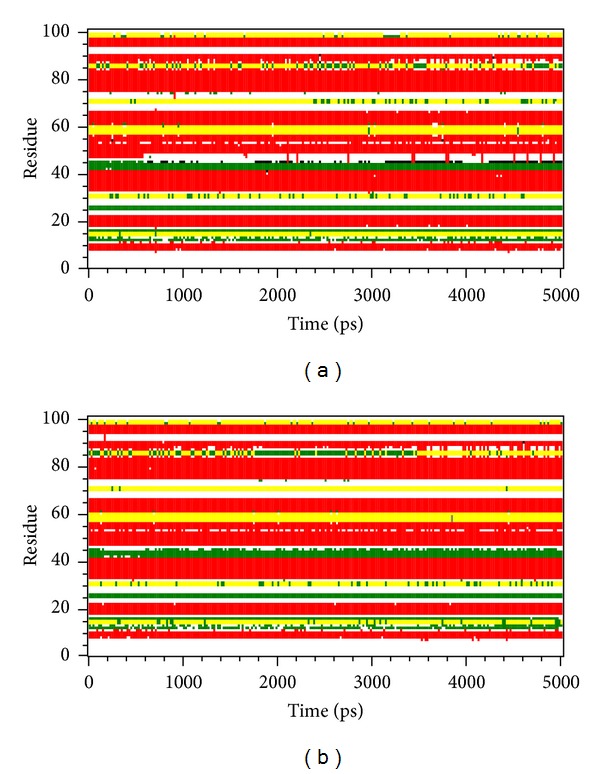
DSSP analysis of (a) protein complex with calycosin and (b) apoprotein.

**Figure 12:**
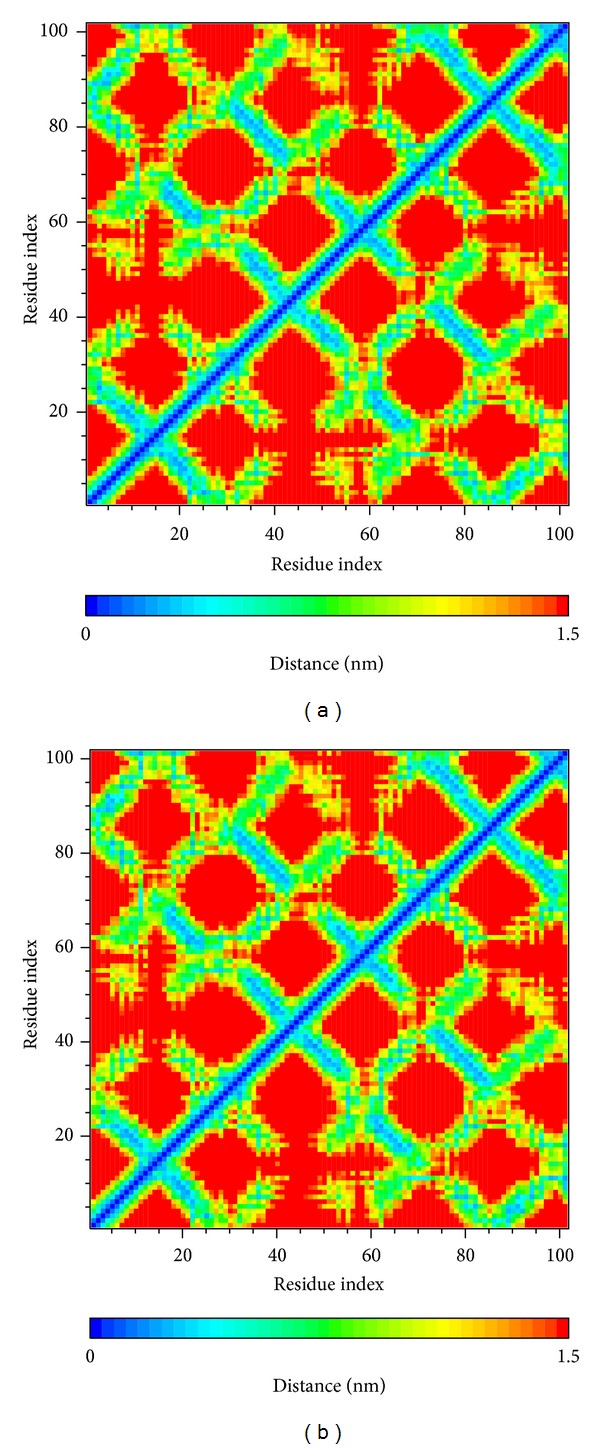
Mdmat analysis of (a) protein complex with calycosin and (b) apoprotein.

**Figure 13:**
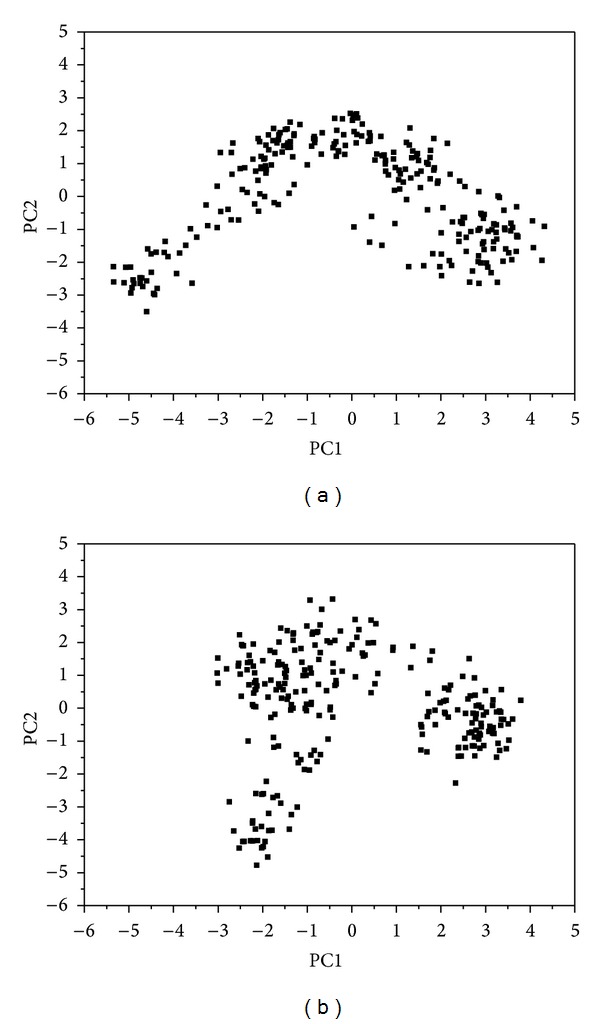
Eigenvector distribution of (a) protein complex with calycosin and (b) apoprotein.

**Figure 14:**
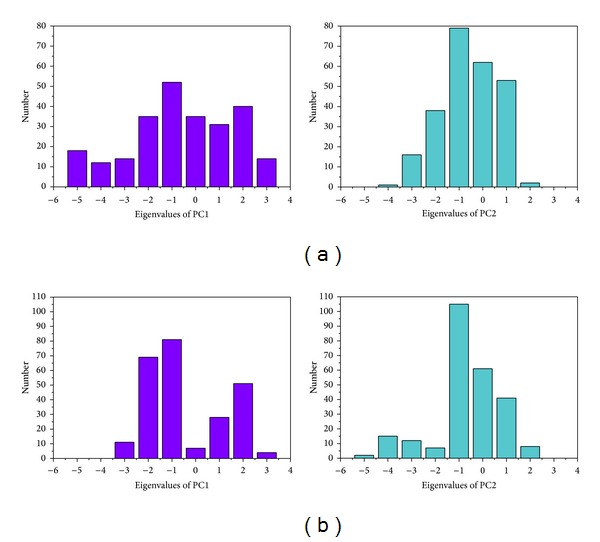
Analysis of eigenvectors PC1 and PC2 for (a) protein complex with calycosin and (b) apoprotein.

**Figure 15:**
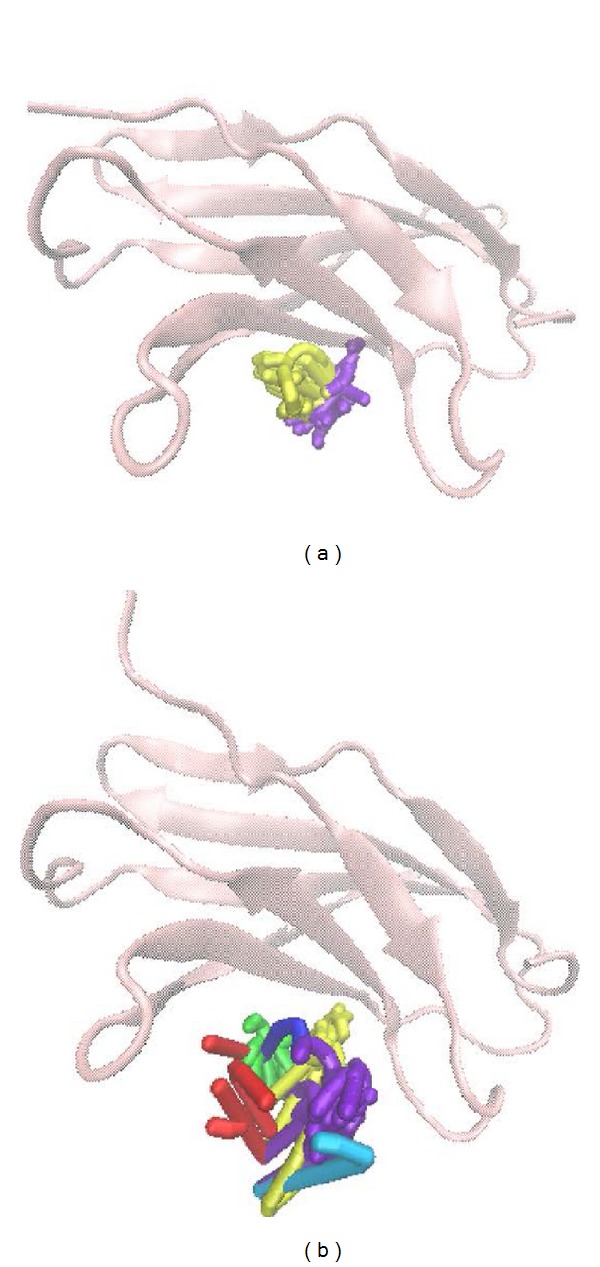
Analysis of transport pathways for (a) protein complex with calycosin and (b) apoprotein.

Figure 5 shows the variation of root-mean-squared deviation (RMSD) for protein complexes with candidate compound and apoprotein in the process of molecular dynamics. For protein RMSD, it indicates the changes of IL6R protein structure for apoprotein and protein complexes with candidate compound. The variation of protein RMSD for apoprotein is more stable than protein complexes with candidate compound during MD simulation. For ligand RMSD, the values of RMSD for candidate compound tend to approximately 0.10–0.15 nm.  Figure 6(a) shows that protein complexes with calycosin had lower gyrate scores than the apoprotein, which indicated that the protein combined with calycosin is more stable than apoprotein. As shown by the slope of MSD in Figure 6(b), the protein combined with calycosin has higher diffusion changes than apoprotein as the slope is increasing after 2 ns, which may have an influence on the protein displacement status. The total energy of protein complexes with candidate compound and apoprotein over 5000 ns MD is located between −367000 and −36000. There is no significant difference between protein complexes with candidate compound and apoprotein (Figure 7). The value of RMSF illustrates the flexibility of each amino acid in a time period of MD simulation.  Figure 8 indicates that the important amino acids Glu144, Gln147, and Ala160 in protein complexes with calycosin are more stable. The clustering analysis can display the representative conformation of protein complexes with calycosin (cutoff of 0.142 nm) (Figure 9(a)) and apoprotein (cutoff of 0.145 nm) (Figure 9(b)).  Figure 10 illustrates the variation of distances between the mass centers of protein and calycosin. It shows that the binding of calycosin is not stable at initial, but it tends to stable after 2000 ps. The structure of DSSP (Figure 11) and the variation of Mdmat distribution (Figure 12) have no significant difference between protein complexes with calycosin and apoprotein.  Figure 13 displays the eigenvector distribution for protein complexes with calycosin and apoprotein. Due to a combination of calycosin, the distribution of eigenvector PC1 has expanded from −2 to −5, and eigenvector PC2 has contracted from −5 to −2, which indicates that there are some changes in the protein structure after combination. In addition, Figure 14 shows the distribution of eigenvectors PC1, PC2 and also illustrated the variation in the distribution of eigenvectors PC1, PC2. The results of transport pathway analysis shown in Figure 15 indicate the presumable pathway of small molecules with colors for protein complexes with calycosin and apoprotein. Protein complex with calycosin has less potential pathway than apoprotein, which indicates that the space of binding domain has variate after a combination of calycosin.

## 4. Conclusion

In this study, we employed the TCM database for virtual screening and ranking the results by the scoring function of Dock score, -PLP1, -PLP2, and H-bond forming residues, H-bond quantity, and Pi forming residues. The influence of top candidate, calycosin, was investigated using apoprotein as the control. After MD simulation, the analysis of RMSD, Gyrate, MSD, total energy, RMSF, cluster, distance of mass centers between protein and calycosin, DSSP, Mdmat, eigenvector, and analysis of transport pathway are performed for investigating the influence of calycosin binding in the receptor. Although there is only slight change in the protein structure, the analysis indicates that protein complex with calycosin is more stable than the apoprotein and the space of binding domain has decreased after a combination of calycosin as there are fewer pathways than apoprotein. Calycosin is known to be one of the components of* Angelica sinensis*, which has been indicated to have an important role in the treatment of rheumatoid arthritis. Therefore, we speculate that calycosin is a potential candidate as lead compounds for further study in drug development process with IL6R protein against rheumatoid arthritis.

## Figures and Tables

**Table 1:** Dock score and other criteria used in screening the TCM database for the top twenty candidates.

Name	Dock score	-PLP1	-PLP2	H-bond forming residues	H-bond quantity	Pi forming residues
Calycosin	43.247	56.29	54.8	Glu144, Gln147, Ala160	3	GLN158
Valerophenone-o-carboxylic_acid	42.473	35.29	34.33	Ala160	2	—
Senkyunolide_D	41.848	39.67	41.1	Glu144	1	—
p-Hydroxyphenethyl_trans-ferulate	41.789	44.6	43.56	Glu144	1	—
Coniferyl_ferulate	41.717	54.16	59	Asn110, Glu144, Gln147, Gln158	5	—
Riligustilide	41.56	48.51	45.81	—	0	—
Ferulic_acid	41.345	48.61	46	Glu144, Ala160	2	—
Angeliferulate	40.976	46.41	40.02	Gln147, Gln158	3	—
Sinaspirolide	40.283	45.76	42.57	—	0	—
Senkyunolide_P	40.067	46.2	41.59	Asn110	1	—
Angelicide	39.634	43.26	43.72	Asn110	1	—
Senkyunolide_H	39.53	46.55	41.92	Glu144, Ala160	5	—
Senkyunolide-I	39.471	34.44	33.41	Glu144, Ala160	3	—
6_7-Ditydroxyligustide	38.932	48.1	50.08	Glu144, Gln147, Gln158	4	—
Vanillic_acid	38.163	36.88	34.7	Glu144, Gln158	2	—
Ononin	38.015	23.01	25.02	Glu144	2	—
Senkyunolide_I	37.198	29.15	34.73	Glu144, Ala160	3	—
3-Butylidene-4-hydro-phthalide	36.452	30.55	29.62	Asn110, Glu144	2	—
Senkyunolide_F	35.047	30.92	36.36	Glu144, Ala160	2	—
Formononetin	34.358	53.75	50.82	Aln147	1	Gln158
